# Single-Cell RNA-seq Identifies Cell Subsets in Human Placenta That Highly Expresses Factors Driving Pathogenesis of SARS-CoV-2

**DOI:** 10.3389/fcell.2020.00783

**Published:** 2020-08-19

**Authors:** Nancy Ashary, Anshul Bhide, Priyanka Chakraborty, Stacy Colaco, Anuradha Mishra, Karisma Chhabria, Mohit Kumar Jolly, Deepak Modi

**Affiliations:** ^1^Molecular and Cellular Biology Laboratory, ICMR-National Institute for Research in Reproductive Health, Indian Council of Medical Research (ICMR), Mumbai, India; ^2^Center for BioSystems Science and Engineering, Indian Institute of Science, Bengaluru, India

**Keywords:** placenta, trophoblast, SARS-CoV-2, coronaviruses, receptors, single-cell RNA-seq, inflammation, COVID-19

## Abstract

Infection by the Severe Acute Respiratory Syndrome-Coronavirus-2 (SARS-CoV-2) results in the novel coronavirus disease COVID-19, which has posed a serious threat globally. Infection of SARS-CoV-2 during pregnancy is associated with complications such as preterm labor and premature rupture of membranes, and a proportion of neonates born to infected mothers are also positive for the virus. During pregnancy, the placental barrier protects the fetus from pathogens and ensures healthy development. To predict if the placenta is permissive to SARS-CoV-2, we utilized publicly available single-cell RNA-seq data to identify if the placental cells express the necessary factors required for infection. SARS-CoV-2 binding receptor *ACE2* and the S protein priming protease *TMPRSS2* are co-expressed by a subset of syncytiotrophoblasts (STB) in the first trimester and extravillous trophoblasts (EVT) in the second trimester human placenta. In addition, the non-canonical receptor *BSG/CD147* and other proteases (*CTSL, CTSB*, and *FURIN*) are detected in most of the placental cells. Other coronavirus family receptors (*ANPEP* and *DPP4*) were also expressed in the first and second trimester placental cells. Additionally, the term placenta of multiple species including humans expressed *ACE2*, *DPP4*, and *ANPEP* along with the viral S protein proteases. The *ACE2*- and *TMPRSS2*-positive (*ACE2* + *TMPRSS2* +) placental subsets expressed mRNA for proteins involved in viral budding and replication. These cells also had the mRNA for proteins that physically interact with SARS-CoV-2 in host cells. Further, we discovered unique signatures of genes in *ACE2* + *TMPRSS2* + STBs and EVTs. The *ACE2* + *TMPRSS2* + STBs are highly differentiated cells and express genes involving mitochondrial metabolism and glucose transport. The second trimester *ACE2* + *TMPRSS2* + EVTs are enriched for markers of endovascular trophoblasts. Both these subtypes abundantly expressed genes in the Toll-like receptor pathway. The second trimester EVTs are also enriched for components of the JAK-STAT pathway that drives inflammation. We carried out a systematic review and identified that in 12% of pregnant women with COVID-19, the placenta was infected with SARS-CoV-2, and the virus was detected in STBs. To conclude, herein we have uncovered the cellular targets for SARS-CoV-2 entry and have shown that these cells can potentially drive viremia in the developing human placenta. Our results provide a basic framework toward understanding the paraphernalia involved in SARS-CoV-2 infections in pregnancy.

## Introduction

Epidemiologic evidence indicates that pregnant women are at higher risk of severe illness and mortality from viral infections such as influenza, Ebola and Lassa fever (Silasi et al., 2015). Certain viral infections during pregnancy can lead to several adverse pregnancy outcomes such as spontaneous abortion, mother-to-child transmission resulting in congenital viral syndromes, still-births and intrauterine fetal deaths (Silasi et al., 2015; Arora et al., 2018). Furthermore, viral infection also predisposes the pregnancy toward preterm birth, which has major long-term health implications for the newborn. Thus, understanding the health risks of viral infections during pregnancy is vital for designing appropriate approaches for its clinical management. The importance of understanding the role of viral infection during pregnancy gains further relevance as we are confronted with newer pandemics, which may affect the pregnant mother and the fetus.

Coronaviruses (CoV) are positive-sense RNA viruses that, upon zoonotic transmission, lead to respiratory disease in humans and some animals. Previous outbreaks of zoonotic coronaviruses, including the severe acute respiratory syndrome (SARS)-CoV and the Middle East respiratory syndrome (MERS)-CoV, have proven to be of great public health concern. Another outbreak of severe acute respiratory syndrome called coronavirus disease-2019 (COVID-19) has been recently reported, which is due to infection by a novel coronavirus termed SARS-CoV-2 (Zhu et al., 2020). The infection has spread rapidly worldwide due to high human-to-human transmission, resulting in a public health emergency of international concern (Bharti et al., 2020; Gupta et al., 2020; Li R. et al., 2020). Presently, there are no specific treatments available for COVID-19, and there is an urgent need to identify the drugs and vaccines targeted against this virus (Prajapat et al., 2020).

Since SARS-CoV-2 has not been detected in humans before, limited information is available about its health effects; negligible information is available for pregnant women. In pregnant women, COVID-19 is associated with severe pregnancy complications such as preterm labor and premature rupture of membranes (Gajbhiye et al., 2020). Furthermore, a proportion of neonates born to mothers with COVID-19 are positive for the virus, suggesting the possibility of vertical transmission through the placental barrier (Gajbhiye et al., 2020; Knight et al., 2020).

The placenta is a highly specialized organ that maintains the equilibrium between immunological and biochemical factors required for fetal development (Deshpande and Balasinor, 2018). It also acts as a barrier for vertical transmission of pathogens (Maltepe and Fisher, 2015; Burton et al., 2016). However, some viruses such as Zika can infect the placental cells via receptors on trophoblasts, leading to fetal malformation and pregnancy complications (Hirsch et al., 2018; Tabata et al., 2018). Interestingly, placental and amniotic fluid infections of SARS-CoV-2 have been reported (Baud et al., 2020; Zamaniyan et al., 2020). For SARS-CoV-2 to be able to infect the placenta, the host cells must harbor the necessary receptors and virus-processing machinery. It has been shown that SARS-CoV-2 binds and infects host cells by utilizing the membrane-bound Angiotensin-Converting Enzyme II (ACE2), which is considered its canonical mode of action (Jagtap et al., 2020; Letko et al., 2020; Shang et al., 2020). In addition, SARS-CoV-2 binds to CD147/Basigin (BSG) on the cell surface that may act as an alternate non-canonical receptor (Wang et al., 2020). Upon receptor binding, the viral-encoded S protein requires cleavage by host proteases for efficient membrane fusion. The main host protease that mediates S protein priming and initiates viral entry is the Type II transmembrane serine protease TMPRSS2 (Hoffmann et al., 2020). The endosomal protease cathepsin L (CTSL) can also enhance viral entry (Hoffmann et al., 2020). Thus, the presence of such receptors and S protein primer proteases in host cells is a key determinant of SARS-CoV-2 infection. Indeed, expression of *ACE2* and *TMPRSS2* have been detected in lung airway cells and the upper respiratory epithelium, the primary site of SARS-CoV-2 action (Sungnak et al., 2020; Ziegler et al., 2020). Beyond respiratory distress, some patients with SARS-CoV-2 viremia develop multiple organ injuries, and cells of these tissues also express *ACE2* and *TMPRSS2* (Qi et al., 2020; Seow et al., 2020; Zou et al., 2020).

The binding of enveloped viruses like SARS-CoV-2 to its receptors results in events related to membrane fusion and/or endocytosis followed by establishment of the primary infection. Following its entry and uncoating, coronavirus replication is initiated by translation of its non-structural proteins including the replicases that allow viral RNA synthesis and capping. This course requires a network of host factors to create an optimal environment for facilitating viral entry, gene expression, RNA synthesis and virus release (de Wilde et al., 2018). Further, most enveloped viruses bud at the plasma membrane by recruiting the host endosomal sorting complex required for transport (ESCRT) machinery (Ahmed et al., 2019; Gatta and Carlton, 2019). While the precise host proteins in SARS-CoV-2 entry and replication are not yet understood, its host interactome has been characterized (Gordon et al., 2020). The host proteins that interact with SARS-CoV-2 are involved in endocytosis and replication of viruses (Gordon et al., 2020). Thus, elucidating tissue and cell-type-specific host machinery that not only mediate viral entry but also replication and budding from the host cell is essential to understand the pathogenesis of SARS-CoV-2 infection.

Single-cell RNA sequencing (scRNA-seq) of different tissues has transformed our ability to map the types, subsets and states of cells in healthy and diseased conditions in an unprecedented manner (Sharma et al., 2018; Szabo et al., 2019; Iyer et al., 2020). Recently, scRNA-seq has been applied to expand our understanding of the cellular landscape during viral infection including that of SARS-CoV-2 (Russell et al., 2018; Galinato et al., 2019; Liao et al., 2020). scRNA-seq has also been used in the identification of various tissues and cells that are potential targets of SARS-CoV-2, and these studies have immensely contributed toward expanding our understanding of the molecular characteristics of the host cells that are targets of viral infection (Colaco et al., 2020; Lukassen et al., 2020; Qi et al., 2020; Seow et al., 2020; Singh et al., 2020; Sungnak et al., 2020; Zhang et al., 2020).

To gain an insight into the pathogenesis of SARS-CoV-2 infection during pregnancy, it is essential to identify and characterize the placental cell types that express the viral receptors ACE2 and BSG/CD147, along with the proteases TMPRSS2 and CTSL. Recent studies have reported *ACE2*-positive cells in early embryonic trophoblasts as well as first trimester human placenta (Colaco et al., 2020; Li M. et al., 2020; Singh et al., 2020). ACE2 protein is also detected in term human placenta^1^. Studies have also shown that *BSG*/*CD147* is expressed in first trimester human trophoblasts and gestational day 18 mouse placenta (Bharadwaj et al., 2011; Lee et al., 2013). However, information regarding the cells co-expressing various coronavirus receptors and S protein proteases as well as their detailed characteristics in the placenta is unknown. Herein (Ashray et al., 2020), we surveyed the publicly available scRNA-seq data of human placenta for the expression of the SARS-CoV-2 receptors *ACE2* and *BSG*/*CD147*, along with the S protein proteases *TMPRSS2* and *CTSL.* We also surveyed the placental cells for the expression of *DPP4* and *ANPEP*, which are utilized by MERS-CoV and CoV-229E, respectively. The study also aimed to characterize the *ACE2*- and *TMPRSS2*-positive placental cells for their possible roles in viral endocytosis, replication, SARS-CoV-2 interactions and viral budding. The results reveal that placental cells are potential targets for SARS-CoV-2 infection.

## Materials and Methods

To identify the population of human placental cells that express *ACE2, BSG, TMPRSS2*, and *CTSL* at single-cell resolution, we analyzed scRNA-seq data of first and second trimester human placenta (Liu et al., 2018) [Accession number GSE89497]. This dataset is derived out of 7 first trimester (8 weeks) placentae and 1 second trimester placenta (24 weeks) in which single cells were isolated by enzymatic digestion followed by enrichment of cells using a combination of methods. Extravillous trophoblasts (EVTs) were enriched by Magnetic Activated Cell Sorting (MACS) using anti-HLA-G antibody, cytotrophoblasts (CTBs) were enriched using anti-CDH1 antibody, and syncytiotrophoblasts (STBs) were manually sorted. The HLA-G- and CDH1-negative fraction was designated to be villous stromal cells (STR). To isolate EVTs from the placenta at 24 weeks, the basal plate was dissected from the villi of the placenta, and the single cells were prepared using enzymatic digestion followed by MACS using anti-HLA-G antibody. We deliberately chose this “index sorted” dataset over the unbiased agnostic scRNA-seq datasets since our major focus was to identify the cellular targets of SARS-CoV-2 specifically in the placenta, and such *a priori* approach allows analysis of homogeneous cell populations and accurate linking of rare transcripts with the index sorted cells.

To understand changes in the expression of *ACE2, BSG, TMPRSS2*, and *CTSL* in the second and third trimester placenta, bulk RNA-seq data was analyzed [Accession number GSE124282]. This dataset is derived out of 4 second trimester and term human placentae (Wang et al., 2019). Bulk RNA-seq data (Armstrong et al., 2017) was also analyzed of term placenta from human, cow, dog, armadillo, elephant, opossum, mouse and bonobo samples [Accession number GSE79121] to understand if the expression of the SARS-CoV-2 receptors in the term placenta is evolutionarily conserved. Pseudo-bulk scRNA-seq data of human first trimester decidua (Suryawanshi et al., 2018) was then analyzed to understand the distribution of *ACE2, BSG, TMPRSS2*, and *CTSL* in different maternal cell populations.

We profiled the mRNA levels of 27 host proteins involved in human ESCRT for viruses (Ahmed et al., 2019) and mRNA levels of 30 proteins involved in viral replication (de Wilde et al., 2018) in placental cells that co-express *ACE2* and *TMPRSS2* in first and second trimester (Supplementary Table 1). We also analyzed the transcript profiles of 332 human proteins that physically interact with SARS-CoV-2 in placental cells that co-express *ACE2* and *TMPRSS2* (Gordon et al., 2020). To guard against viral infection, host cells express a plethora of genes that sense the presence of the virus on the cell surface, in cytosol and in endosomes. This in turn activates the host defense mechanisms to limit or eliminate viral infection and restore tissue homeostasis. We profiled the expression of 487 host viral response genes in placental cells that co-express *ACE2* and *TMPRSS2* (Supplementary Table 1).

To characterize the trophoblast cells that co-express *ACE2* and *TMPRSS2* as well as their counterparts that do not express both of these genes; we carried out pseudo-bulk analysis of *ACE2*- and *TMPRSS2*-positive (*ACE2* + *TMPRSS2* +) and *ACE2*- and *TMPRSS2*-negative (*ACE2*–*TMPRSS2*–) trophoblast cells. Single-cell data for *ACE2* + *TMPRSS2* + and *ACE2–TMPRSS2–* cells was independently aggregated and the mean TPM values were computed. The data was filtered for genes whose mean values were ≥0.1 TPM and the ratio of the mean value in *ACE2* + *TMPRSS2* + cells over *ACE2*–*TMPRSS2*– cells was calculated. The genes that had a ratio of ≥1.5 or ≤0.5 and *p*-value of <0.05 were filtered and the data was deconvoluted for single cells. Gene ontology (GO) analysis was performed using the PANTHER database and over-representation tests were performed using reference genes of PANTHER pathways^2^.

To determine if SARS-CoV-2 is detected in the placenta, one author (AB) carried out a systematic review with the keywords “placenta and SARS-CoV-2,” “COVID-19 and placenta,” “SARS-CoV-2 and pregnancy,” “coronaviruses” and “pregnancy.” The primary outcome was to determine the number of cases reporting the presence of SARS-CoV-2 in placental tissue; the secondary outcome was to determine the placental cell types positive for SARS-CoV-2. Searches were carried out in PubMed, Google Scholar, MedRxiv, bioRxiv and other preprint databases. Articles reporting primary data in which detection of SARS-CoV-2 was carried out by either RT-PCR and/or by immunohistochemistry or electron microscopy were included. Reviews, blog and newspaper reports were excluded. Data was entered in tabular format and was independently verified by another author (DM).

All the data was processed using R Studio version 3.6.2. Heatmaps were plotted using the heatmap.2 function from the gplots R package and the pheatmap R package. Uniform Manifold Approximation and Projection (UMAP) analysis was performed using the UMAP 0.2.6.0 R package. To identify the cell clusters, 500 genes with high SD and average log2-transformed expression >1 were selected. Next, these 500 genes were given as an input to UMAP for calculating the projections of all cells. Statistical analysis was done using the Welch’s *t*-test and the graphs were plotted in GraphPad Prism version 8.0.

## Results

### Trophoblast Cells Express mRNA for SARS-CoV-2 Receptors and Spike Protein Processing Enzymes

**FIGURE 1:**
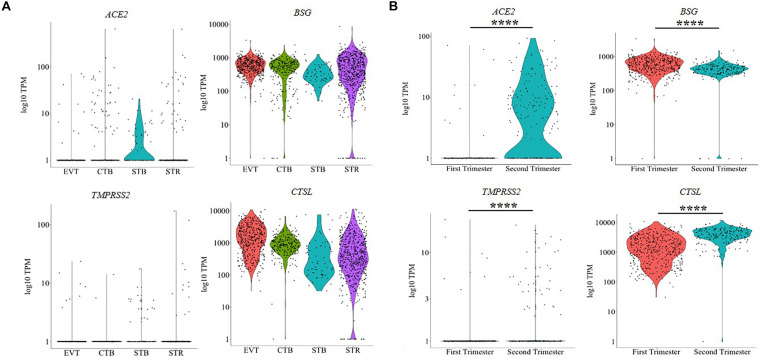
Trophoblast cells express mRNA for SARS-CoV-2 receptors and spike protein processing enzymes. **(A)** mRNA level of SARS-CoV-2 receptors (*ACE2* and *BSG*) and spike protein primer enzymes (*TMPRSS2* and *CTSL*) in different cell types of first trimester human placenta [EVT (*n* = 440), CTB (*n* = 248), STB (*n* = 64), STR (*n* = 615)]. **(B)** Comparison of the mRNA levels of *ACE2, BSG, TMPRSS2*, and *CTSL* in EVT of first (*n* = 440 cells) and second (*n* = 200 cells) trimester human placenta. Each dot represents data of a single cell, the *Y* axis represents log_10_ Transcripts Per Million (TPM). Bar within the cluster denotes mean. Horizontal black bars denote significantly different values (**** indicates *p*-value ≤ 0.0001). Data was extracted from single-cell RNA-seq of human placenta (Liu et al., 2018) [Accession number GSE89497]. EVT, extravillous trophoblast; CTB, cytotrophoblast; STB, syncytiotrophoblast; STR, villous stromal cell.

The human placenta is characterized by four distinct cell lineages: extravillous trophoblasts (EVT), cytotrophoblasts (CTB), syncytiotrophoblasts (STB), and villous stromal cells (STR). To understand the distribution of the SARS-CoV-2 receptors in these cell types, we analyzed publicly available single-cell transcriptome data from human placenta. The results revealed that *ACE2*, *BSG*, *TMPRSS2* and *CTSL* were expressed in all the cell types of first trimester placenta; however, not every cell of each type expressed these genes (Figure 1A). As evident, STBs represented the highest proportion of *ACE2*-expressing cells (39%) in first trimester placenta, whereas only 2% of EVTs had *ACE2* expression. *BSG* was abundantly expressed in almost all the cells (96–100%) of EVT, CTB, STB, and STR of the first trimester (Supplementary Table 2). Very few cells of the human placenta expressed *TMPRSS2.* The highest *TMPRSS2* expression was detected in STBs of first trimester placenta (23%), whereas only 1% of CTBs had *TMPRSS2* expression (Supplementary Table 2). *CTSL* was expressed in nearly all STBs, CTBs, EVTs and STRs of first trimester placenta (Figure 1A). The numbers of cells that express these genes individually are given in Supplementary Table 2.

We then compared the expression of these genes in EVTs of the first trimester and second trimester human placenta (Figure 1B). 2% of first trimester EVTs and 62% of second trimester EVTs expressed *ACE2*. Similarly, the numbers of EVTs expressing *TMPRSS2* also increased in the second trimester as compared to the first trimester (2 vs. 19%) (Supplementary Table 2). In both cases, the increase was statistically significant (*p*-value ≤ 0.001). Nearly all EVT cells in the first and the second trimesters expressed *BSG* and *CTSL* (Supplementary Table 2). *BSG* expression was significantly reduced in second trimester EVTs as compared to the first trimester (*p*-value ≤ 0.001); the expression of *CTSL* was significantly higher in second trimester EVTs as compared to first trimester EVTs (*p*-value ≤ 0.001) (Figure 1B).

We also studied the expression of other SARS-CoV receptors *DPP4* and *ANPEP*, which are utilized by MERS-CoV and CoV-229E, respectively. *ANPEP* was detected in the EVTs, CTBs and STRs but not in the STBs of the first trimester placenta. *DPP4*, on the other hand, was expressed by all CTBs, STBs, EVTs, and STRs (Supplementary Figure 1A). As compared to first trimester EVTs, the levels of *ANPEP* significantly decreased (*p*-value ≤ 0.001), while those of *DPP4* significantly increased (*p*-value ≤ 0.001) in the second trimester EVTs (Supplementary Figure 1B).

Bulk transcriptome analysis revealed that, in comparison to the second trimester, the levels of *ACE2, BSG*, and *DPP4* were reduced in the term placenta; however, a statistically significant reduction was observed only in the case of *BSG* (*p*-value ≤ 0.05). Although the levels of *TMPRSS2* and *ANPEP* were higher in the term placenta as compared to second trimester placenta, this increase was not statistically significant. *CTSL* levels were similar in the second trimester and term placentae (Supplementary Figure 2A). In the absence of publicly available index sorted scRNA-seq data of term placenta, we cannot comment on the cell types that express these genes.

The expression of coronavirus receptors and spike protein proteases was compared in term placenta of different species (Supplementary Figure 2B). *ACE2* transcripts were detected in the placenta of most mammals except bonobo. *BSG* mRNA was expressed in term placenta of all the species, albeit at varying levels. *TMPRSS2* mRNA was expressed in the placenta of most species except dog, armadillo, and elephant. *CTSL* mRNA was detected in human, cow, dog, mouse, and bonobo placentae. *ANPEP* was only expressed in human, armadillo and mouse placentae, and *DPP4* was expressed in placenta of all species except mouse.

To determine if endometrial cells express these genes, we analyzed pseudo-bulk data of the first trimester feto-maternal interface (Supplementary Figure 3). Expression of *ACE2* was detected in smooth muscle cells, and a low abundance of *ACE2* transcripts was also detected in decidual stromal cells and fibroblasts, vascular endothelial cells and NK cells. *TMPRSS2* transcripts were detected in endometrial epithelial cells and lymphatic endothelial cells. *BSG* and *CTSL* were detected in all the maternal cells of the first trimester feto-maternal interface.

### Co-expression of mRNA of SARS-CoV-2 Receptors and Spike Protein Processing Enzymes in Human Placental Cells

**FIGURE 2:**
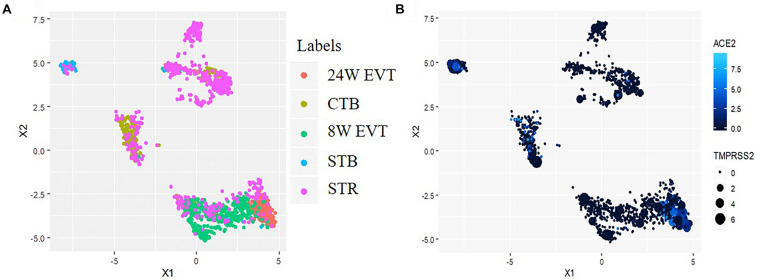
Uniform Manifold Approximation and Projection (UMAP) plot illustrating cell clusters and *ACE2* & *TMPRSS2* expressing cells in the human placenta. Data was extracted from single-cell RNA-seq of human placenta (Liu et al., 2018) [Accession number GSE89497]. **(A)** Clusters consist of three trophoblast lineages (EVT, extravillous trophoblast; CTB, cytotrophoblast; STB, syncytiotrophoblast) and the villous stromal cells (STR). **(B)** The points colored blue are the cells co-expressing *ACE2* and *TMPRSS2*. In each blue point, the intensity of the color represents *ACE2* expression and the size of the point represents the extent of *TMPRSS2* expression.

Uniform Manifold Approximation and Projection revealed distinct clusters of CTBs and STBs, and EVTs in the first trimester placenta; the second trimester EVTs also formed an independent cluster (Figure 2A). Some STR cells clustered independently while others clustered with the different trophoblast cell types, suggesting that the STRs are not a pure population. This finding was expected since the STRs were the post-enrichment leftover fractions of CTBs, STBs and EVTs. The data on this population was hence excluded in further analysis. SARS-CoV-2 infection in host cells requires coordinated expression of the entry receptor *ACE2* and S protein primer *TMPRSS2*. UMAP analysis revealed that a subset of CTBs, STBs and EVTs co-expressed both *ACE2* and *TMPRSS2* (Figure 2B).

**FIGURE 3:**
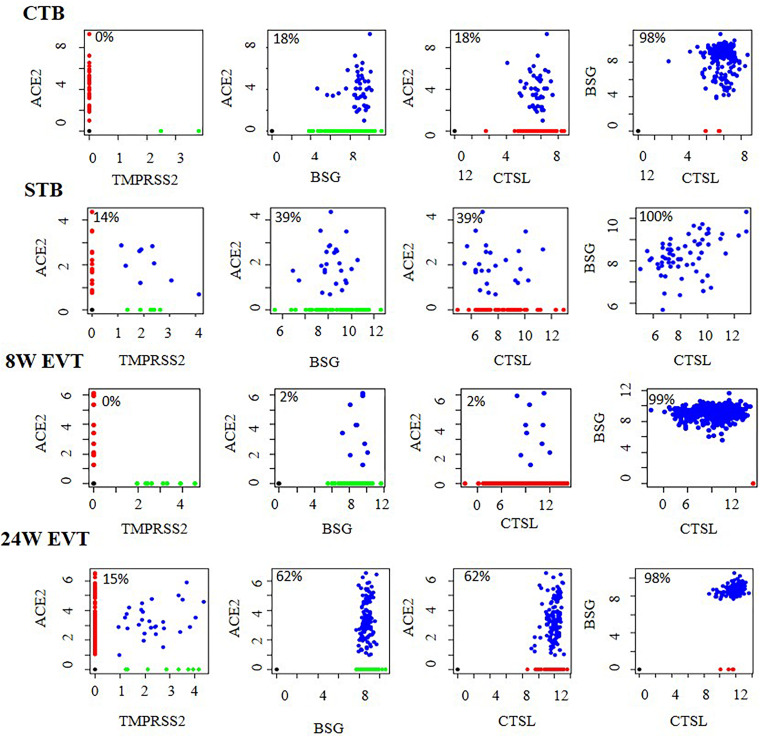
Co-expression of mRNA of SARS-CoV-2 receptors and spike protein processing enzymes in human placental cells. Co-expression of *ACE2* and *TMPRSS2*, *ACE2* and *BSG, ACE2* and *CTSL*, and *BSG* and *CTSL* in STB (*n* = 64), CTB (*n* = 248), first trimester EVT (8W EVT, *n* = 440) and second trimester EVT (24W EVT, *n* = 200). Each dot represents data of a single cell. Values in each box are the percentage of co-expressing cells. Co-expressing cells are blue; single-positive cells are red and green. X and Y axes represent log_2_ Transcripts Per Million (TPM) values for that gene. Data was extracted from single cell RNA-seq of human placenta (Liu et al., 2018) [Accession number GSE89497]. EVT, extravillous trophoblast; CTB, cytotrophoblast; STB, syncytiotrophoblast.

We next evaluated CTBs, STBs and EVTs co-expressing *ACE2, TMPRSS2*, *BSG*, and *CTSL* in different combinations (Figure 3). The results revealed that a subset of STBs (14%) in the first trimester placenta co-expressed *ACE2* and *TMPRSS2* (Supplementary Table 3). No other cell types in first trimester placenta expressed this receptor and S protein primer protease pair, although there were cells expressing *ACE2* (Figure 3). However, 15% of EVTs in the second trimester placenta co-expressed *ACE2* and *TMPRSS2* (Supplementary Table 3).

In the first trimester placenta, all the *ACE2*-positive trophoblast subtypes co-expressed *BSG* and *CTSL*, and all the *BSG*-positive cells co-expressed *CTSL*. All the *ACE2*-positive second trimester EVTs co-expressed *BSG* and *CTSL*, and all the *BSG*-positive cells co-expressed *CTSL* (Figure 3). The absolute numbers and percentages of the co-expressing cells are given in Supplementary Table 3.

### *ACE2* + *TMPRSS2* + First Trimester Syncytiotrophoblast Cells Are Highly Differentiated and Express the Machinery for Viral Endocytosis, Replication and Budding

Since only STBs co-expressed *ACE2* and *TMPRSS2* in the first trimester, we carried out an in-depth characterization of these cells. 14% of the total STB population of the first trimester placenta expressed both *ACE2* and *TMPRSS2*, 52% did not express either, and the rest of the cells expressed either *ACE2* or *TMPRSS2* (Supplementary Table 3). We compared the expression profiles of classical STB genes between the *ACE2* and *TMPRSS2* co-expressing cells and *ACE2-* and *TMPRSS2-*negative STBs. We observed that both cell types abundantly expressed the transcripts for human chorionic gonadotropin beta 5 (*CGB5*) and somatomammotropin [placental lactogen (*CSH1*)], as well as steroid hormone biosynthesis enzymes (*HSD17B1* and *CYP19A1*). Further, both these subsets of STBs abundantly expressed the other putative SARS-CoV-2 S protein primers *FURIN* and Cathepsin B (*CTSB*) (Supplementary Figure 4).

**FIGURE 4:**
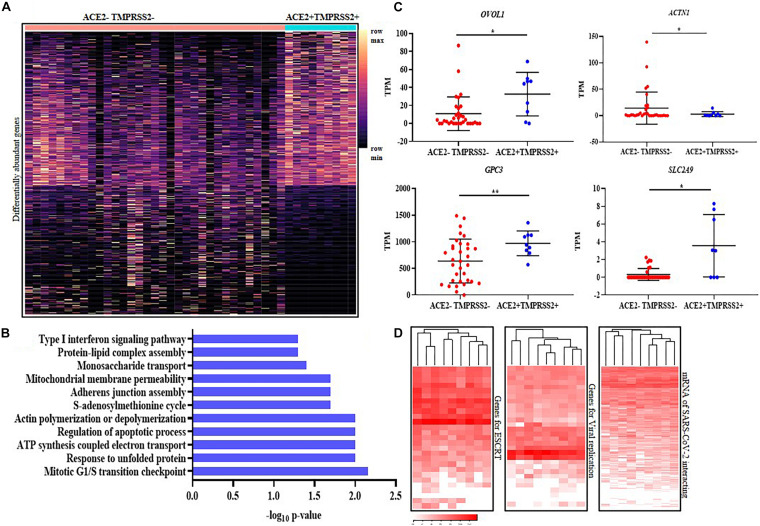
*ACE2* + *TMPRSS2* + first trimester syncytiotrophoblast cells are terminally differentiated and express the machinery for viral endocytosis, replication and budding **(A)** Distribution of 817 differentially expressed genes in *ACE2-* and *TMPRSS2*-positive (*n* = 9) (*ACE2* + *TMPRSS2* +) and *ACE2-* and *TMPRSS2*-negative (*ACE2– TMPRSS2*–) cells (*n* = 33) of the first trimester syncytiotrophoblast. Rows represent genes and columns represent individual cells, presented on a relative color scale. **(B)** Biological processes enriched in the differentially expressed genes of the first trimester syncytiotrophoblast. The *Y* axis indicates the enriched biological processes and *X* axis is the -log_10_ of the raw *p*-values. **(C)** mRNA levels of *OVOL1*, *GPC3, SLC2A9*, and *ACTN1* in *ACE2–TMPRSS2*– and *ACE2* + *TMPRSS2* + cells. Each dot represents data of a single cell, the *Y* axis represents Transcripts Per Million (TPM). Bars denote mean ± SD. Horizontal black bars denote significantly different values (* indicates *p*-value ≤ 0.05, ** indicates *p*-value ≤ 0.001). **(D)** Heatmap showing the expression of genes involved in endosomal sorting complexes required for transport (ESCRT), replication and host genes involved in SARS-CoV-2 interaction. In all heat maps, each row depicts a gene and each column depicts a single *ACE2-* and *TMPRSS2*-positive (*n* = 9) (*ACE2* + *TMPRSS2* +) cell in the first trimester syncytiotrophoblast. The data is presented on a relative color scale in which the minimum and maximum values in each row are used to convert values to colors. Data was extracted from single cell RNA-seq of human placenta (Liu et al., 2018) [Accession number GSE89497].

We next characterized the transcriptome differences between the *ACE2* + *TMPRSS2* + versus the *ACE2*–*TMPRSS2*– STB cells. Pseudo-bulk analysis identified 817 genes (including *ACE2* and *TMPRSS2*) between these two cell types (Supplementary Table 4). Of these, 444 were over represented while the others were under represented in the *ACE2* + *TMPRSS2* + cells as compared to *ACE2*–*TMPRSS2*– cells. These genes were heterogeneously expressed in the *ACE2*–*TMPRSS2*– STBs, while most *ACE2* + *TMPRSS2* + cells uniformly expressed these genes (Figure 4A). The biological processes enriched by these genes included regulation of G1/S cell-cycle checkpoints, actin polymerization/depolymerization, regulation of mitochondrial membrane permeability and electron-transport-coupled ATP synthesis, monosaccharide transport and unfolded protein response (Figure 4B). Most of the *ACE2* + *TMPRSS2* + cells significantly overexpressed the transcription factor *OVOL1* (a terminal STB differentiation marker), and the glucose transport regulators *GPC3* and *SLC2A9* (*p*-value ≤ 0.05). The expression of *ACTN1* (an actin binding protein) was significantly downregulated in *ACE2* + *TMPRSS2* + versus *ACE2–TMPRSS2–* STBs (*p*-value ≤ 0.05) (Figure 4C).

We next analyzed the mRNA levels of 27 genes involved in human ESCRT of viruses and 30 host genes involved in SARS-CoV replication in *ACE2-* and *TMPRSS2*-positive STB cells. All the *ACE2* + *TMPRSS2* + STBs uniformly expressed most of these genes (Figure 4A); however, the other cells showed heterogeneous expression across the different subtypes (Supplementary Figure 5). We also analyzed the mRNA levels of 332 host proteins that are known to interact with SARS-CoV-2 and found that there was minimal heterogeneity in expression of these genes in the first trimester STBs (Figure 4D) as compared to other cell types (Supplementary Figure 5).

### Second Trimester *ACE2* + *TMPRSS2* + Cells Are Invasive Extravillous Trophoblasts and Express Markers of Endovascular Trophoblasts

Amongst the second trimester EVTs, 15% of cells were *ACE2* + *TMPRSS2* + while 33% did not express either of the transcripts (Supplementary Table 3). Two populations of second trimester EVTs are reported and characterized by the expression of TAC3. Type 1 EVTs are TAC3-high and express genes involved in migration and invasion; type 2 EVTs are TAC3-low cells that express genes involved in cell proliferation (Liu et al., 2018). In addition to TAC3, the type 1 EVTs also express JAM2, SERPENIN1 and PRG2 (Liu et al., 2018). We observed that the levels of *TAC3* were marginally but not significantly higher in *ACE2* + *TMPRSS2* + EVTs (Supplementary Figure 6A), and the mRNA levels of other genes were identical in *ACE2* + *TMPRSS2* + EVTs as compared to cells not expressing either of the two genes (*ACE2– TMPRSS2–*) (Supplementary Figure 6A). Principal component analysis did not reveal major differences in the transcriptome of the *ACE2* + *TMPRSS2* + and *ACE2*–*TMPRSS2*– cells (Supplementary Figure 6B). We compared the expression profiles of classical EVT genes between the *ACE2* + *TMPRSS2* + and *ACE2*–*TMPRSS2*– cells and observed that both the cell types abundantly expressed the transcripts for *HLA-G* and *ITGB1* (Supplementary Figure 6C). Both these subsets of EVTs also abundantly expressed other SARS-CoV-2 S protein primer proteins *FURIN* and *CTSB* of which the levels of *FURIN* were significantly higher (*p*-value ≤ 0.05) in *ACE2* + *TMPRSS2* + EVTs as compared to *ACE2*–*TMPRSS2*– EVTs (Supplementary Figure 6C).

**FIGURE 5:**
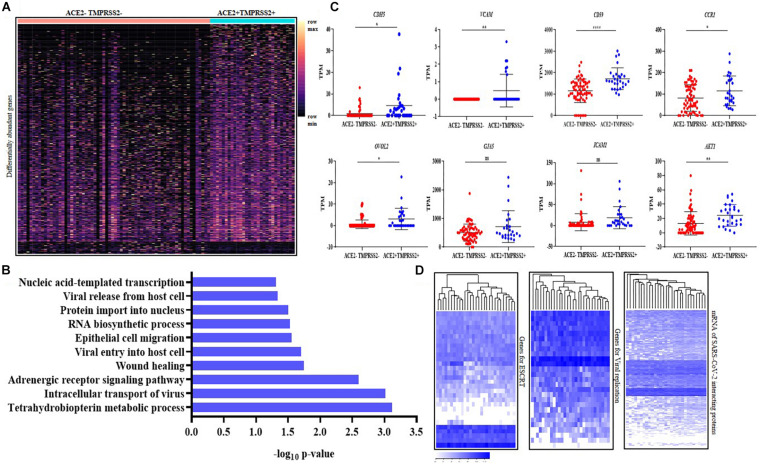
Second trimester *ACE2* + *TMPRSS2* + cells are invasive extravillous trophoblasts and express markers of endovascular trophoblasts. **(A)** Distribution of 983 differentially expressed genes in *ACE2-* and *TMPRSS2*-positive (*n* = 29) (*ACE2* + *TMPRSS2* +) and *ACE2-* and *TMPRSS2*-negative (*n* = 66) (*ACE2–TMPRSS2*–) cells of the second trimester extravillous trophoblast. Rows represent genes and columns represent individual cells, presented on a relative color scale. **(B)** Biological processes enriched in the *ACE2* + *TMPRSS2* + cells (*n* = 29) of the second trimester extravillous trophoblast. The *Y* axis indicates the enriched biological processes and *X* axis is the enrichment score. **(C)** Comparison of mRNA level of *CDH5, VCAM, CCR1, CD59, OVOL2, ICAM*, and *AKT1* in *ACE2–TMPRSS2*- (*n* = 66) and *ACE2* + *TMPRSS2* + (*n* = 29) cells. Each dot represents data of a single cell, the *Y* axis represents Transcripts Per Million (TPM). Bars denote mean ± SD. Horizontal black bars denote significantly different values (* indicates *p*-value ≤ 0.05, ** indicates *p*-value ≤ 0.001, **** indicates *p*-value < 0.0001). **(D)** Heatmap showing the expression of genes involved in endosomal sorting complexes required for transport (ESCRT), replication and host genes involved in SARS-CoV-2 interaction. In all heat maps, each row depicts a gene and each column depicts a single *ACE2* + *TMPRSS2* + cell of the second trimester extravillous trophoblast. The data is presented on a relative color scale in which the minimum and maximum values in each row are used to convert values to colors. Data was extracted from single cell RNA-seq of human placenta (Liu et al., 2018) [Accession number GSE89497].

To characterize if there are any specific classes of genes differentially abundant between the *ACE2* + *TMPRSS2* + versus the *ACE2*–*TMPRSS2*– EVT cells, pseudo-bulk analysis was carried out. There were 983 differentially abundant genes (including *ACE2* and *TMPRSS2*) between these two cell types (Supplementary Table 5) of which 931 were overrepresented and 52 were underrepresented in the *ACE2* + *TMPRSS2* + cells as compared to *ACE2*–*TMPRSS2*– cells. Further, these genes were heterogeneously expressed in the *ACE2*–*TMPRSS2*– EVTs while most *ACE2* + *TMPRSS2* + cells uniformly expressed these genes (Figure 5A). Most of these differentially abundant genes enriched several GO biological processes such as viral entry, release and intracellular transport. The other enriched GO biological processes were nucleic acid replication, epithelial morphogenesis and cell migration (Figure 5B).

The *ACE2* + *TMPRSS2* + cells significantly overexpressed the markers of endovascular trophoblasts *CDH5, VCAM*, *CCR1* and *CD59* (*p*-value ≤ 0.05) (Figure 5C). These cells also significantly overexpressed *OVOL2*, the marker of terminally differentiated EVTs, and the invasion-related marker *AKT1* (*p*-value ≤ 0.05) (Figure 5C). *ICAM* and *GJA5* are markers for EVTs in anchoring cell columns, and their levels were identical in the *ACE2* + *TMPRSS2* + and the *ACE2*–*TMPRSS2*– EVT cells.

Analysis of the mRNA levels of 27 genes involved in human ESCRT of viruses and 30 host genes involved in SARS-CoV replication in *ACE2-* and *TMPRSS2*-positive cells at single-cell resolution revealed that all the *ACE2* + *TMPRSS2* + EVTs uniformly expressed most of these genes (Figure 5D), while the first trimester EVTs that had no *ACE2* + *TMPRSS2* + cells had a very heterogeneous expression of these genes (Supplementary Figure 5). We also analyzed the mRNA levels of 332 host proteins that interact with SARS-CoV-2 and observed that almost all these genes were expressed in most *ACE2* + *TMPRSS2* + second trimester EVTs (Figure 5D), while the first trimester EVTs had heterogeneous expression (Supplementary Figure 5).

### Unique Signatures of Genes Involved in Viral Response in First Trimester Syncytiotrophoblast Cells and Second Trimester Extravillous Trophoblasts

**FIGURE 6:**
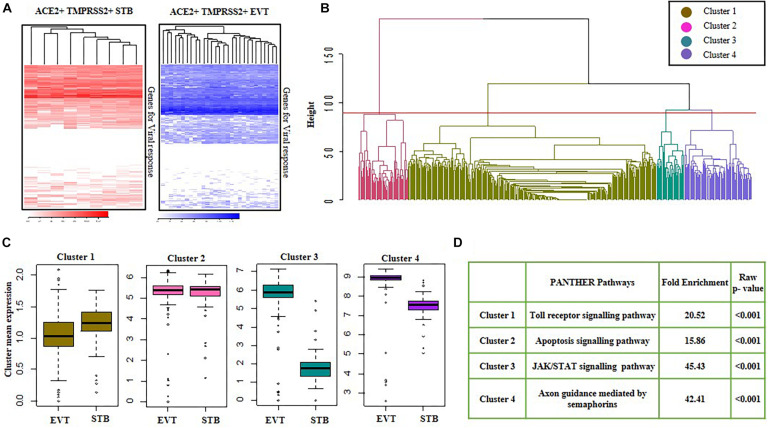
Viral response genes in first trimester syncytiotrophoblasts and second trimester extravillous trophoblasts. **(A)** Heatmap showing the expression of genes involved in viral response in *ACE2-* and *TMPRSS2*-positive (*ACE2* + *TMPRSS2* +) cells of first trimester STB (*n* = 9) and second trimester EVT (*n* = 29). Each row is a gene and each column represents data for a single *ACE2* and *TMPRSS2* co-expressing cell. **(B)** Cluster dendrogram of first trimester STB and second trimester EVT cells. The horizontal axis of the dendrogram represents the distance or dissimilarity between clusters. The vertical axis represents the clusters. **(C)** Mean of four clusters of upregulated genes in first trimester STB and second trimester EVT cells. *Y* axis represents cluster mean expression and *X* axis represents different cell types. **(D)** Pathways enriched in the in four clusters of first trimester STBs and second trimester EVTs, with their enrichment score and *p*-values. EVT, extravillous trophoblast; STB, syncytiotrophoblast. Data was extracted from single cell RNA-seq of human placenta (Liu et al., 2018) [Accession number GSE89497].

We studied the baseline expression of 487 genes involved in viral response in both first trimester STBs and second trimester EVTs (Supplementary Table 1). Only a subset of viral response genes were expressed in *ACE2* + *TMPRSS2* + EVTs and STBs (Figure 6A). The heatmaps showed minimal heterogeneity across cells but high variability in expression across genes involved in the viral response. To characterize these genes in EVT and STB cells, genes were clustered by a hierarchical clustering method using the “hclust” function available in R. Genes were grouped based on the most optimum threshold, which resulted in four different gene clusters (Figure 6B). The average of the gene expression in Clusters 1 and 2 were identical in both EVTs and STBs. However, average expression of genes in Clusters 3 and 4 was more abundant in EVTs as compared to STBs (Figure 6C).

Next, GO analysis was performed using the PANTHER database for all four gene clusters. For each cluster, an over-representation test was performed using reference genes of PANTHER pathways, and a pathway with the highest fold-enrichment value was selected as the enriched pathway for a given gene cluster. GO classification of these clusters revealed that most of the genes in Cluster 1 had a role in the Toll-like receptor (TLR) signaling response and the genes in Cluster 2 had a role in apoptosis. Additionally, Cluster 3, which included genes of the JAK-STAT pathway, and Cluster 4, which included genes for axon guidance mediated by semaphorins, were enriched in EVTs as compared to STBs (*p*-value ≤ 0.05) (Figure 6D).

### SARS-CoV-2 Infects the Human Placenta and Is Localized in Syncytiotrophoblast Cells

To determine if SARS-CoV-2 can infect human placenta, a systematic review was carried out (Supplementary Table 6). Seventeen studies that reported analysis of SARS-CoV-2 in placental tissue from 93 pregnant women with COVID-19 were identified. In these studies, SARS-CoV-2 was detected by reverse transcriptase PCR (RT-PCR), immunohistochemistry or electron microscopy. Of the 93 placentae, 5 were second trimester placentae and 88 were term/preterm placentae. In all, 12% of placentae were reported to be positive for SARS-CoV-2. Second trimester placentae from all 5 women with COVID-19 were positive for SARS-CoV-2 while term/preterm placenta from ∼7% of women with COVID-19 had SARS-CoV-2 positivity. Immunohistochemistry of 1 second trimester and 2 term placentae revealed the presence of SARS-CoV-2 protein in STBs. Viral particles were also identified in STBs of second trimester and preterm (28 weeks) placenta by electron microscopy (Supplementary Table 6).

## Discussion

Herein, we utilized scRNA-seq to identify and characterize the potential cellular targets of SARS-CoV-2 infection in human placenta. To review the data presented: (1) The mRNA for coronavirus receptors (*ACE2, BSG, DPP4*, and *ANPEP*) and the S protein proteases (*TMPRSS2* and *CTSL*) are expressed in the first trimester to term placenta, (2) The SARS-CoV-2 binding receptor *ACE2* and the S protein priming protease *TMPRSS2* are co-expressed by a subset of syncytiotrophoblasts (STB) in the first trimester and extravillous trophoblasts (EVT) in the second trimester human placenta, (3) These *ACE2* + *TMPRSS2* + subsets are highly differentiated STBs and endovascular EVTs, (4) The *ACE2* + *TMPRSS2* + placental subsets readily express mRNA for proteins involved in ESCRT of viruses and replication, in addition to transcripts for proteins that are known to interact with SARS-CoV-2 structural and non-structural proteins, and (5) The STBs and EVTs differentially express genes involved in the host response to viral infection.

Using a scRNA-seq dataset (Vento-Tormo et al., 2018), *ACE2*-positive CTBs and STBs are reported in the first trimester human placenta (Li M. et al., 2020; Singh et al., 2020). Corroborating these findings using a different dataset (Liu et al., 2018), we show the presence of *ACE2* in CTBs and STBs of first trimester placenta. However, along with STBs and CTBs, we also identified *ACE2* expression in EVTs of the first and second trimester placenta, which has not been reported earlier. In addition to *ACE2*-expressing STBs and EVTs, our study revealed that *BSG/CD147*, the alternate receptor for SARS-CoV-2 (Wang et al., 2020), is expressed by almost all the placental cells. We also detected abundant expression of *DPP4* (the receptor for MERS-CoV) and *ANPEP* (the receptor for CoV-229E) in the cells of the placenta. Like *ACE2*, *DPP4* was detected in all the cell types of the first trimester placenta and also in EVTs of the second trimester; very few STBs expressed *ANPEP.* Similar observations are made using different datasets of scRNA-seq of first trimester human placenta (Pique-Regi et al., 2020; Singh et al., 2020). In addition to the first and second trimester placenta, *ACE2, TMPRSS2, BSG, ANPEP*, and *DPP4* transcripts are also detected in human term placenta by bulk RNA-seq. However, we cannot comment on the cell types that express these genes due to the lack of publicly available scRNA-seq datasets of term human placenta. Interestingly, these genes are also expressed in the term placenta of different species including mice, cows, dogs, armadillos, elephants, opossums and bonobos. Thus, we propose that multiple cell types in the placental tissue could be targets of different coronaviruses throughout gestation.

While *ACE2* is the primary receptor for SARS-CoV-2 entry, the S protein of SARS-CoV-2 undergoes cleavage by a cell surface protease, *TMPRSS2* (Hoffmann et al., 2020). Whether *ACE2* and *TMPRSS2* are required on the same cell to activate SARS-CoV-2 S protein to invade *ACE2* single-positive cells is a matter of investigation. However, as active S protein has a finite lifetime (Shulla et al., 2011), its processing at the plasma membrane will make it most effective for viral entry. Thus, we assumed that for SARS-CoV-2, the *ACE2* and *TMPRSS2* co-expressing cells would have the highest infectivity. Our analysis revealed that a proportion of STBs (14%) in the first trimester and a subset of EVTs (15%) in second trimester human placenta co-express *ACE2* and *TMPRSS2*. Contradicting this proposition, Pique-Regi et al. (2020) reported that co-expression of *ACE2* and *TMPRSS2* is negligible in the trophoblasts of human first, second and third trimester placenta. Differences in the methods of tissue sampling, cell isolation and inefficiencies in detection of low-abundance transcripts in scRNA-seq can underestimate the actual frequencies of *ACE2* + cells in a given tissue. Indeed, the dataset used in this study is exclusively of MACS enriched trophoblast preparations while the datasets used by Pique-Regi et al. (2019, 2020) are a mixed population of cells from the feto-maternal interface. It is known that fractionated cell preparations allow better identification of low abundance transcripts in rare cell populations (Nguyen et al., 2018). These factors could be the possible reasons that our analysis could identify more numbers of *ACE2* and *TMPRSS2* co-expressing cell types in the human placenta. While the numbers of placental cells co-expressing *ACE2* and *TMPRSS2* may appear insignificant considering the total placental volume, it must be borne in mind that only 3–6% of lung airway epithelial cell subtypes (the primary site of SARS-CoV-2 action) co-express both *ACE2* and *TMPRSS2* (Ziegler et al., 2020).

Beyond the canonical *ACE2* and *TMPRSS2* based entry, SARS-CoV-2 also utilizes *BSG*/*CD147* as the non-canonical mode of entry (Wang et al., 2020). Presently, the mechanism by which *BSG*/*CD147* mediates viral entry in host cells is unknown. In other cells, *BSG*/*CD147* promotes entry of viruses by endocytosis (Pushkarsky et al., 2001). It is possible that the same mechanism may be operative in the case of SARS-CoV-2. In this context, it is interesting that all the *BSG/CD147*-positive cells abundantly co-expressed the endosomal protease *CTSL.* Further, we observed that almost all the *ACE2* + STBs and EVTs co-expressed *BSG/CD147*, suggesting that more than one mechanism may operate for viral entry in these cells of the human placenta. Beyond *TMPRSS2*, studies have identified that SARS-CoV-2 may have a *FURIN* cleavage site, leading to a broader set of host proteases that could mediate S protein priming (Coutard et al., 2020). The *ACE2* + *TMPRSS2* + STBs and EVTs abundantly express *FURIN* as well as another endosomal protease, *CTSB*. Together our data conclusively show that multiple cells of human placenta are targets for SARS-CoV-2 binding and entry with S protein priming by both canonical and non-canonical pathways.

We next aimed to characterize the placental cells that are potential targets for SARS-CoV-2 infection. As the *ACE2*-mediated viral entry is a well-established mechanism, we focused only on characterizing the STBs of the first trimester and EVTs of the second trimester placenta that co-express both *ACE2* and *TMPRSS2* in a proportion of cells while others are devoid of these transcripts. In the developing placenta, trophoblast stem cells differentiate into cytotrophoblasts, which undergo further differentiation to form the non-self-renewing cytotrophoblasts, extravillous trophoblasts and syncytiotrophoblasts (Turco and Moffett, 2019; Hemberger et al., 2020). The syncytiotrophoblasts covering the villi are major hormone secreting cells and function as a protective immunological barrier (Maltepe and Fisher, 2015; Gupta et al., 2016; Liu et al., 2018; Vento-Tormo et al., 2018; Turco and Moffett, 2019). We observed that STBs that co-express both *ACE2* and *TMPRSS2* also express the mRNA for the peptide hormones and enzymes for steroid hormone biosynthesis. However, their levels are not significantly different from their *ACE2*–*TMPRSS2*– counterparts, suggesting that both these cell types retain the basic functions of STBs. However, pseudo-bulk analysis revealed that the *ACE2* + *TMPRSS2* + cells are enriched for genes involved in cell cycle checkpoints, actin filament remodeling, mitochondrial functions, hexose transport and type I interferon signaling. Indeed, the terminally differentiated STBs have replicative senescence and require extensive cytoskeletal remodeling for syncytialization; the mitochondria of STBs play a key role in progesterone synthesis by providing cholesterol (Martinez et al., 2015). Additionally, these cells are enriched in *OVOL1*, the transcription factor required for STB specification, as well as proteins involved in glucose transport across the feto-maternal barrier, a key function of well differentiated STBs (Jansson and Ylve, 2002; Renaud et al., 2015; Vento-Tormo et al., 2018; Turco and Moffett, 2019). These results imply that the *ACE2* + *TMPRSS2* + cells are a subset of highly differentiated STBs and these cells are potential targets for viral entry. Indeed, SARS-CoV-2 mRNA, protein and virions are detected in STBs of second trimester and term/preterm placenta from a woman with COVID-19 (Hosier et al., 2020). Additionally, increased syncytiotrophoblastic knots are observed in placenta from pregnant women with COVID-19 (Chen et al., 2020), which is suggestive of injury to the STBs in the placenta.

We next probed the second trimester EVTs, 15% of which co-express *ACE2* and *TMPRSS2*. The EVTs differentiate from cytotrophoblast stem cells and populate the tips of the placental villi to form the anchoring villi, thus defining the boundary between mother and fetus. The EVTs are central to placentation as they invade into the maternal decidua and are involved in remodeling of maternal spiral arteries, veins and lymphatic ducts (Sharma et al., 2016; Pollheimer et al., 2018). We observed that the *ACE2* + *TMPRSS2* + cells abundantly express the classical EVT marker *ITGB1* and also *HLA-G* that induces tolerogenic immune responses leading to acceptance of the semi-allogeneic fetus. Two kinds of EVTs are reported in the second trimester human placenta: the proliferative EVTs in the cell columns and the invasive endovascular or interstitial EVTs (Pollheimer et al., 2018; Turco and Moffett, 2019), and both of these have a unique transcript signature (Liu et al., 2018). Herein, we observed that while the levels of *TAC3* and other molecules associated with columnar versus invasive EVTs are not significantly different between *ACE2* + *TMPRSS2* + and *ACE2*–*TMPRSS2*– second trimester EVTs, the double-positive cells overexpressed key invasive EVT markers such as *OVOL2, GJA5, ICAM* and *AKT1* (Sharma et al., 2016; Bai et al., 2018; Liu et al., 2018; Jeyarajah et al., 2020), suggesting that these cells are invasive trophoblasts. We further observed that many of the *ACE2* + *TMPRSS2* + cells were enriched for genes having a role in cell migration. The EVTs can either invade the decidua (designated as interstitial EVTs) or remodel the spiral arteries (designated as endovascular EVTs). While both these EVTs are invasive in nature, they have differential expression of certain marker genes. For example, endovascular EVTs overexpress the CDH5 and VCAM, they also have higher expression of CCR1 and CD59 (Bulla et al., 2005; Cartwright and Balarajah, 2005; Liu et al., 2018; Ueda et al., 2019; Sato, 2020). Intriguingly, the *ACE2* + *TMPRSS2* + cells also overexpressed several of the key endovascular EVT markers including *CDH5, CCD5, CD59*, and *VCAM*, indicating that the *ACE2* + *TMPRSS2* + population of second trimester EVTs are potentially endovascular trophoblasts and are targets of SARS-CoV-2 infection. Indeed, pseudo-bulk analysis of the *ACE2* + *TMPRSS2* + cells and *ACE2*–*TMPRSS2*– cells revealed significant enrichment of genes with GO terms involving regulation of viral release from host cells. Most of the *ACE2* + *TMPRSS2* + endovascular EVTs abundantly expressed most genes whose protein products in the host are known to be involved in human endocytosis and budding of viruses and replication. Together, this data shows that SARS-CoV-2 may affect the invading EVTs at the feto-maternal interface in the second trimester and can result in damaged vasculature. In this context, it is important to note that the maternal endothelial cells in the decidua also express *ACE2* and *BSG*, making the maternal endothelium another entry point of SARS-CoV-2 infection at the feto-maternal interface. Any impairment in functions of these cells can cause placental damage and vertical transmission of the virus. Indeed, increased intervillous and subchorionic fibrin deposition and fetal thrombotic vasculopathy with zones of avascular fibrotic villi are observed in placenta of women infected with coronaviruses including SARS CoV-2 (Ng et al., 2006; Baud et al., 2020; Hosier et al., 2020; Mulvey et al., 2020). Together, these results indicate that the integrity of endovascular trophoblasts and the endothelial compartment of the feto-maternal interface may be compromised in women with SARS-CoV-2 infection.

Once the virus binds to its receptors on host cells and gains entry, it utilizes a plethora of host genes for its replication. Post replication, most enveloped viruses complete their life-cycle by forming vesicles that bud from the plasma membrane via the cellular ESCRT (endosomal sorting complexes required for transport) machinery. Interestingly, we observed that the *ACE2* + *TMPRSS2* + STBs and EVTs were enriched for the key genes that encode for proteins involved in ESCRT and viral replication. Using affinity-purification mass spectrometry, 332 human proteins that interact with SARS-CoV-2 have been identified, and many of these play a role in ESCRT and viral replication (Gordon et al., 2020). We observed that *ACE2* + *TMPRSS2* + STBs and EVTs abundantly expressed most of these genes. Thus SARS-CoV-2 may hijack proteins in the EVTs and STBs thereby interfering with normal placental functions. In this context, it is important to highlight that a significant proportion of the human SARS-CoV-2 interacting proteins also interact with proteins of other viruses including Zika and Hepatitis C virus, which replicate in the trophoblast cells (Giugliano et al., 2015; Tabata et al., 2016, 2018; Gordon et al., 2020). Together our data strongly implies that first trimester STBs and second trimester EVTs are not just targets for SARS-CoV-2 entry, but also the virus may be potentially pathogenic to these cells.

A proportion of SARS-CoV-2 proteins target the components of innate immune signaling pathways, including NF-kappa-B (Gordon et al., 2020). We decided to probe this in detail by profiling the STBs and EVTs for 487 genes whose protein products are involved in viral response in host cells. We observe that only a proportion of these genes are expressed in most EVTs and STBs. Based on their expression levels in the host cells, they could be classified in four clusters, and interestingly, the first trimester STBs and the second trimester EVTs expressed genes in the TLR signaling pathway, the primary response to viral infection. Previous studies in SARS-CoV have identified involvement of TLR pathways in protection against viral response (Dosch et al., 2009; Totura et al., 2015). However, we found that the genes in the JAK-STAT pathway were overexpressed in the EVTs but not in the STBs. However, this is not surprising as the JAK-STAT pathway is required for physiological functions of EVTs, namely invasion (Fitzgerald et al., 2010; Suman et al., 2013; Sharma et al., 2016; Godbole et al., 2017). The JAK-STAT pathway is also central for mounting a host response to viral infection, and treatment with interferon gamma induces the expression of interferon-stimulated genes in EVT cells (Verma et al., 2020). Thus, EVTs are not only the entry sites for SARS-CoV-2 infection, but they also possess the cellular machinery to mount an inflammatory response toward an infection. With regard to coronaviruses (including SARS-CoV-2), an overexuberant inflammatory response is observed even at lower viral titres, which contributes to the viral pathogenicity in the lung (Liao et al., 2020). Further, the *ACE2* receptors are induced by interferon signaling in the lung (Ziegler et al., 2020), thereby amplifying the infectious cycle in host tissues. Whether or not a similar mechanism is operative in placental cells is under investigation, but the heightened baseline expression of the JAK-STAT pathway genes in the EVTs itself could readily lead to placental inflammation that may be detrimental to pregnancy. Infiltration of leukocytes and chorioamnionitis is observed in placenta from women with coronavirus infection (Ng et al., 2006; Baud et al., 2020). Since inflammation of the feto-maternal interface causes preterm births (Silasi et al., 2015; Surve et al., 2016), it is plausible that the increased incidence of preterm delivery in women with COVID-19 could be linked to this process.

Beyond preterm births, the demonstration that the *ACE2* + *TMPRSS2* + subpopulation of EVTs consists of invasive endovascular trophoblasts is clinically relevant in conditions like preeclampsia. The invasion of the trophoblast cells and remodeling of the spiral arteries deep into the myometrium is essential for normal fetal growth and development (Norwitz, 2006; Soares et al., 2015). If the arteries are not sufficiently remodeled, there is disordered perfusion of blood and an inadequate supply of nutrients and oxygen, resulting in fetal growth restriction, stillbirth, preeclampsia, placental abruption and preterm labor (Brosens et al., 2019). Since SARS-CoV-2 and other coronaviruses may target the endovascular trophoblasts, it is plausible that the infection could lead to other adverse pregnancy outcomes. Indeed, higher incidence of preeclampsia, preterm labor, fetal distress and premature rupture of membranes are reported in pregnant women infected with SARS-CoV-2 in the third trimester (Gajbhiye et al., 2020). Also, high rates of miscarriages, preterm birth and premature rupture of membranes have been reported for other human coronavirus infections (Alfaraj et al., 2019; Mullins et al., 2020). These observations imply that SARS-CoV-2 infection is detrimental to pregnancy due the possible infection of placental cells such as EVTs.

To determine whether SARS-CoV-2 can infect the placental cells, we carried out a systematic review to identify studies that report presence or absence of the virus in placenta of women with COVID-19. The results revealed that ∼12% (11/93) of placentae obtained from mothers with COVID-19 had detectable levels of SARS-CoV-2 RNA (Supplementary Table 6). Viral RNA, non-structural proteins and intact virions are detected in STBs of the second trimester and preterm/term placenta. This is definitive evidence of placental infection by SARS-CoV-2. However, this may not be an accurate estimate of the frequency of placental infection as most studies included women at term with unknown viral loads and duration of infection. Nevertheless, the fact that some studies have also detected the virus in the amniotic fluid and fetal membranes (Baud et al., 2020; Zamaniyan et al., 2020) as well as IgM in fetal blood (Zeng et al., 2020) implies that the placenta does get infected and the virus can cross the transplacental barrier to infect the fetus. In a systematic review and primary data from a large cohort of pregnant women, mother-to-child transmission of the virus is observed in 5–8% of cases (Gajbhiye et al., 2020; Knight et al., 2020). Thus, it appears likely that the placenta is not just permissive to viral entry but can be a site of active viremia that can lead to the breakthrough of SARS-CoV-2 infection from mother to fetus.

To summarize, this is the first in-depth survey to identify the cellular basis of SARS-CoV-2 infection in the human placenta. However, this data is limited by the constraints of scRNA-seq which includes host and environmental factors that may affect the expression of receptors and proteases. Experimental variations like sites of tissue collection, cell isolation techniques and statistical cut-offs may lead to inclusion or exclusion of specific cell types. Furthermore, our observations need to be corroborated for protein expression. Nevertheless, our results provide a basic framework in understanding of the paraphernalia involved in SARS-CoV-2 infections in pregnancy. It will be essential to determine how SARS-CoV-2 infection alters the temporal dynamics of host responses at the single-cell resolution in the placenta. We believe that this work will aid in developing rational strategies for management of COVID-19 and other coronavirus infections in pregnancy.

## Data Availability Statement

All datasets presented in this study are included in the article/Supplementary Material.

## Ethics Statement

Ethical review and approval was not required for the study on human participants in accordance with the local legislation and institutional requirements. Written informed consent for participation was not required for this study in accordance with the national legislation and the institutional requirements.

## Author Contributions

NA, AB, and PC analyzed and interpreted the data and prepared the figures. SC and AM were involved in data analysis and preparing the manuscript. KC prepared the figures and edited the manuscript. DM conceived the idea and planned this study. DM and MJ spearheaded the study and were involved in data interpretation. All authors were involved in manuscript writing and approved the final version.

## Conflict of Interest

The authors declare that the research was conducted in the absence of any commercial or financial relationships that could be construed as a potential conflict of interest.

## Footnotes

1 https://www.proteinatlas.org/ENSG00000130234-ACE2/tissue/placenta

2 http://www.pantherdb.org/
