# Supplementary figures and images for: Single-Cell RNA-seq Identifies Cell Subsets in Human Placenta That Highly Expresses Factors Driving Pathogenesis of SARS-CoV-2
Source: Front Cell Dev Biol. 2020 Aug 19;8:783. doi: 10.3389/fcell.2020.00783 (PMC7466449; doi:10.3389/fcell.2020.00783)

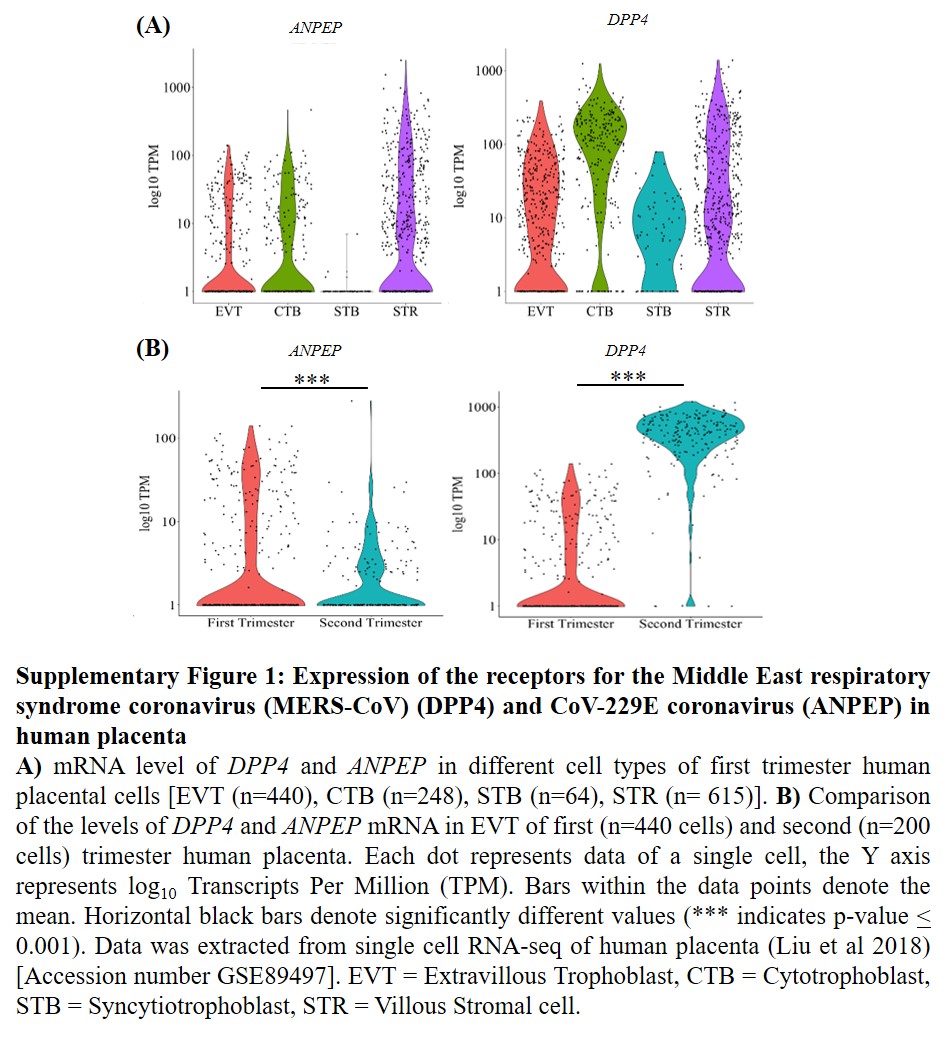

Supplement: Supplementary file 1 [file Image_1.jpg]

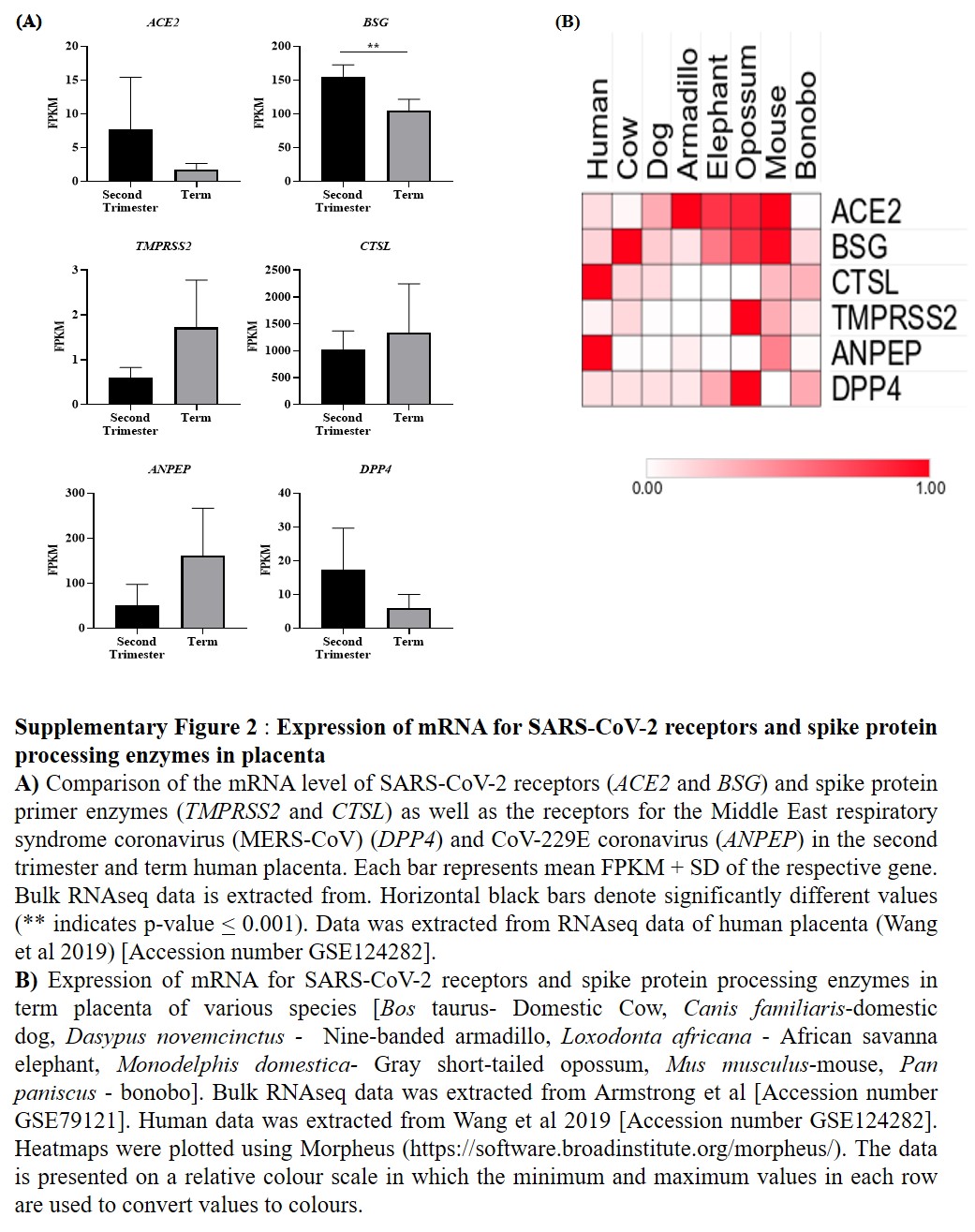

Supplement: Supplementary file 2 [file Image_2.jpg]

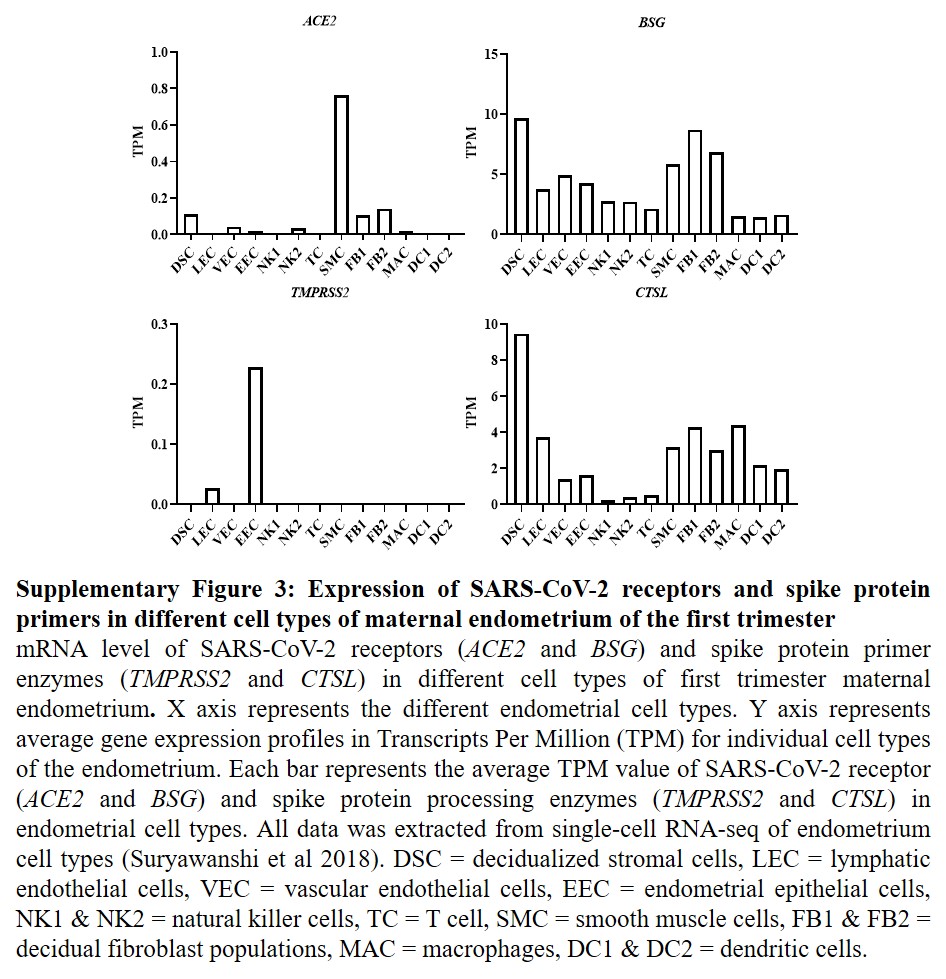

Supplement: Supplementary file 3 [file Image_3.jpg]

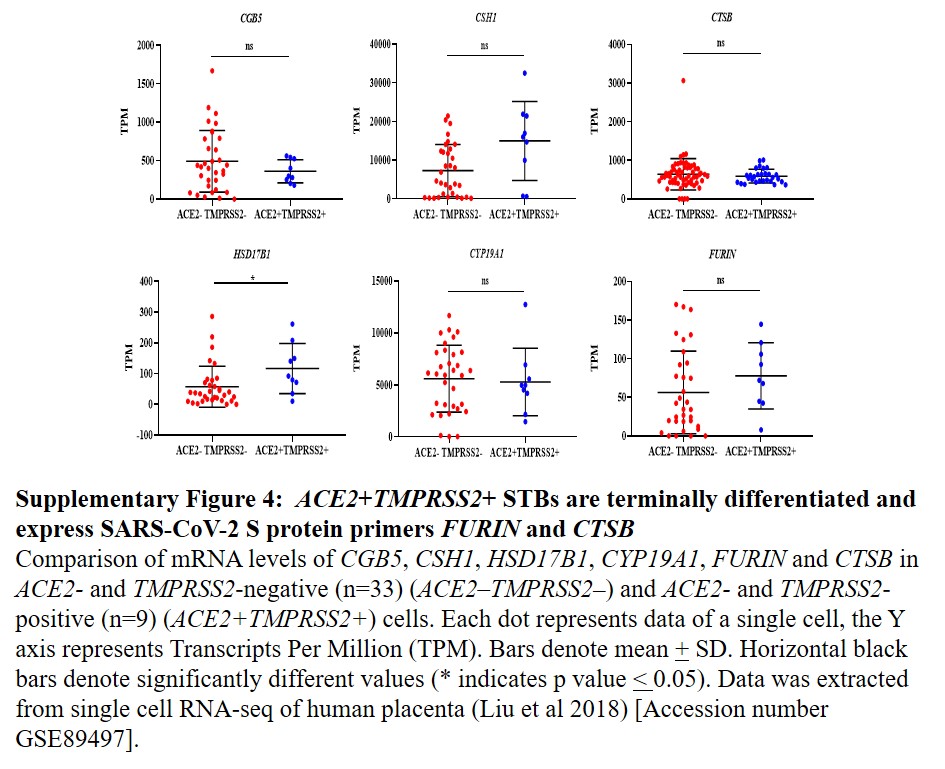

Supplement: Supplementary file 4 [file Image_4.JPEG]

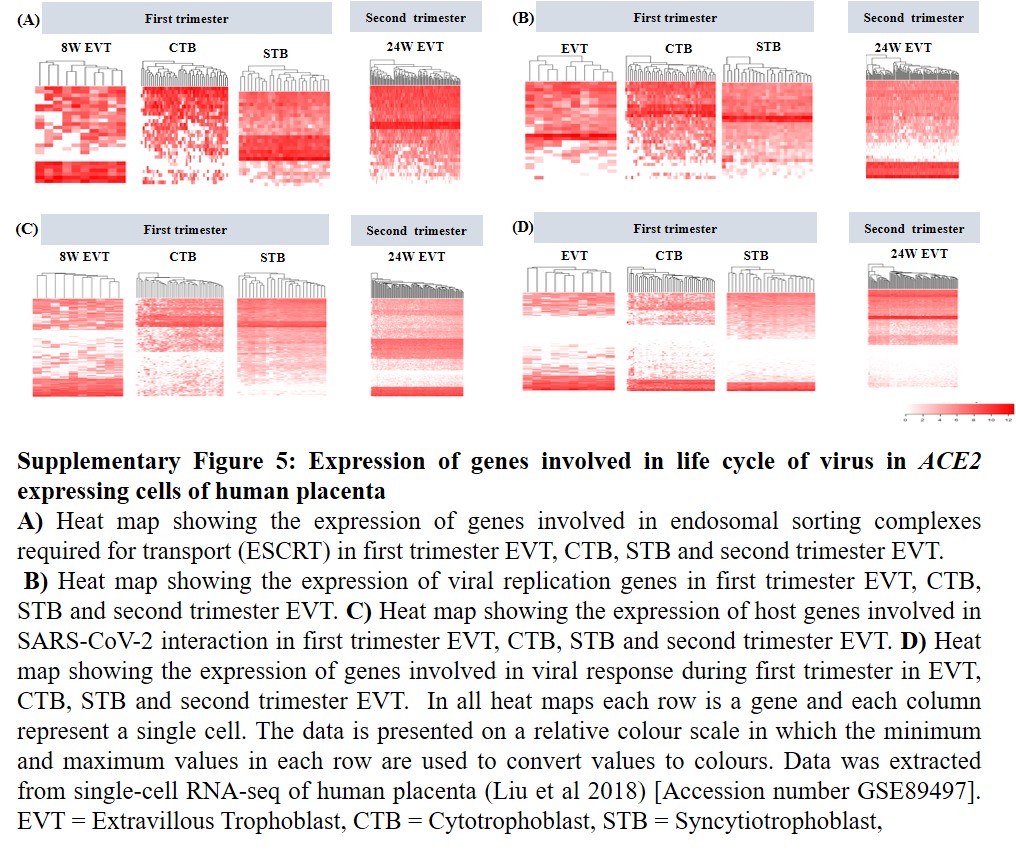

Supplement: Supplementary file 5 [file Image_5.jpg]

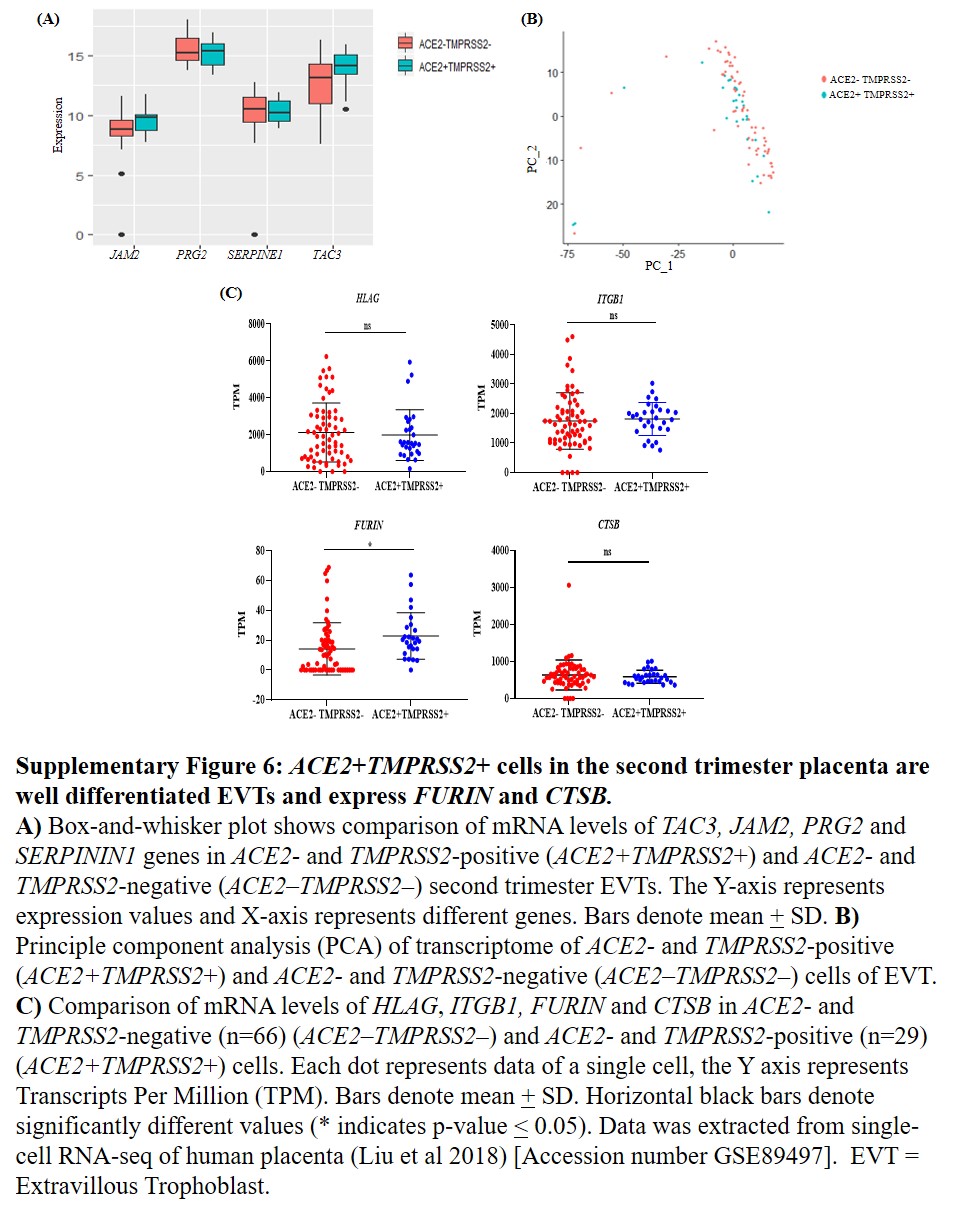

Supplement: Supplementary file 6 [file Image_6.JPEG]
