# Supplementary material for: Single-Cell RNA-seq Identifies Cell Subsets in Human Placenta That Highly Expresses Factors Driving Pathogenesis of SARS-CoV-2
Source: Front Cell Dev Biol. 2020 Aug 19;8:783. doi: 10.3389/fcell.2020.00783 (PMC7466449; doi:10.3389/fcell.2020.00783)
Supplement: Supplementary file 8 [file Table_2.DOCX]

| **Cell type** | **Total cells** | **ACE2+** | **BSG+** | **TMPRSS2+** | **CTSL+** |
| --- | --- | --- | --- | --- | --- |
| **First trimester EVT** | 440 | 10 (2%) | 439 (99%) | 8 (2%) | 440 (100%) |
| **Second trimester EVT** | 200 | 125 (62%) | 195 (97%) | 38(19%) | 199 (99%) |
| **STB** | 64 | 25 (39%) | 64 (100%) | 15(23%) | 64 (100%) |
| **CTB** | 248 | 45 (18 %) | 244 (98%) | 2 (1%) | 247 (99%) |
| **STR** | 615 | 33 (5%) | 593 (96%) | 13 (2%) | 591 (96%) |

**Supplementary table 2:** Percentage of cells expressing SARS-CoV-2 receptors and its spike protein processing enzymes. EVT = Extravillous Trophoblast, CTB = Cytotrophoblast, STB = Syncytiotrophoblast, STR= Villous Stromal Cell.
