# Supplementary material for: Single-Cell RNA-seq Identifies Cell Subsets in Human Placenta That Highly Expresses Factors Driving Pathogenesis of SARS-CoV-2
Source: Front Cell Dev Biol. 2020 Aug 19;8:783. doi: 10.3389/fcell.2020.00783 (PMC7466449; doi:10.3389/fcell.2020.00783)
Supplement: Supplementary file 9 [file Table_3.DOCX]

| **Cell type** | **Total cells** | **ACE2+ BSG+** | **ACE2+ BSG-** | **ACE2+ TMPRSS2+** | **ACE2+ TMPRSS2-** | **ACE2+ CTSL+** | **CTSL+ BSG+** | **ACE2- TMPRSS2-** |
| --- | --- | --- | --- | --- | --- | --- | --- | --- |
| **First trimester EVT** | 440 | 10 (2%) | 0 | 0 | 10 (2%) | 10 (2%) | 439 (99%) | 422 (96%) |
| **Second trimester EVT** | 200 | 125 (62%) | 0 | 29 (15%) | 96 (48%) | 125 (62%) | 195 (98%) | 66 (33%) |
| **STB** | 64 | 25 (39%) | 0 | 9 (14%) | 16 (25%) | 25 (39%) | 64 (100%) | 33 (52%) |
| **CTB** | 248 | 45 (18%) | 0 | 0 | 45 (18%) | 45 (18%) | 244 (98%) | 201 (81%) |
| **STR** | 615 | 33 (5%) | 0 | 1 (0.1%) | 32 (5%) | 33 (5%) | 576 (94%) | 570 (93%) |

**Supplementary Table 3:** Percentage of cells co-expressing SARS-CoV-2 receptors and its spike protein processing enzymes. EVT = Extravillous Trophoblast, CTB = Cytotrophoblast, STB = Syncytiotrophoblast, STR= Villous Stromal Cell.
