# Supplementary material for: Single-Cell RNA-seq Identifies Cell Subsets in Human Placenta That Highly Expresses Factors Driving Pathogenesis of SARS-CoV-2
Source: Front Cell Dev Biol. 2020 Aug 19;8:783. doi: 10.3389/fcell.2020.00783 (PMC7466449; doi:10.3389/fcell.2020.00783)
Supplement: Supplementary file 12 [file Table_6.DOCX]

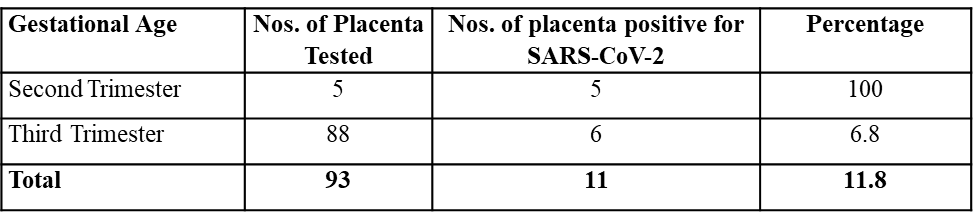


Percentage of tested samples at Second & Third trimester and its prevalence

**(A)**

**(B)**

| **AUTHORS** | **Gestational age of placenta** | **No. of Placenta tested** | **No. of Placenta tested Positive** | **Method of Testing** | **Cell Types positive** | **DOI** |
| --- | --- | --- | --- | --- | --- | --- |
| Patanè, et al. | Term | N=2 | 2 | RT-PCR & IHC | STB | [10.1016/j.ajogmf.2020.100145](https://doi.org/10.1016/j.ajogmf.2020.100145) |
| Hosier et al | 22 Week | N=1 | 1 | IHC, ISH & EM | STB | [10.1172/JCI139569](https://doi.org/10.1172/JCI139569) |
| Gabriela et al | 28 week | N=1 | 1 | EM | STB | 10.1016/j.ajog.2020.05.023 |

**Supplementary Table 6: Prevalence and cellular localization of SARS-CoV-2 in human placenta.**

**A)** Systematic review of 17 studies (references given below) showing prevalence of SARS-CoV-2 in second and third trimester human placenta. **(B)** List of studies showing localization of SARS-CoV-2 in the placental tissue from pregnant women with COVID-19. STB= syncytiotrophoblast, RT-PCR= Real time PCR, IHC= Immunohistochemistry, EM= Electron Microscopy, ISH =RNA in situ hybridization

| 1. Patanè, L., Morotti, D., Giunta, M. R., Sigismondi, C., Piccoli, M. G., Frigerio, L., Mangili, G., Arosio, M., & Cornolti, G. (2020). Vertical transmission of COVID-19: SARS-CoV-2 RNA on the fetal side of the placenta in pregnancies with COVID-19 positive mothers and neonates at birth. *American journal of obstetrics & gynecology MFM*, 100145. Advance online publication. <https://doi.org/10.1016/j.ajogmf.2020.100145> 2. Hosier, H., Farhadian, S. F., Morotti, R. A., Deshmukh, U., Lu-Culligan, A., Campbell, K. H., Yasumoto, Y., Vogels, C. B., Casanovas-Massana, A., Vijayakumar, P., Geng, B., Odio, C. D., Fournier, J., Brito, A. F., Fauver, J. R., Liu, F., Alpert, T., Tal, R., Szigeti-Buck, K., Perincheri, S., … Lipkind, H. S. (2020). SARS-CoV-2 infection of the placenta. *The Journal of clinical investigation*, 139569. Advance online publication. <https://doi.org/10.1172/JCI139569> 3. Algarroba, G. N., Rekawek, P., Vahanian, S. A., Khullar, P., Palaia, T., Peltier, M. R., Chavez, M. R., & Vintzileos, A. M. (2020). Visualization of SARS-CoV-2 virus invading the human placenta using electron microscopy. *American journal of obstetrics and gynecology*, 10.1016/j.ajog.2020.05.023. Advance online publication. <https://doi.org/10.1016/j.ajog.2020.05.023> 4. Penfield, C. A., Brubaker, S. G., Limaye, M. A., Lighter, J., Ratner, A. J., Thomas, K. M., Meyer, J., & Roman, A. S. (2020). Detection of SARS-COV-2 in Placental and Fetal Membrane Samples. *American journal of obstetrics & gynecology MFM*, 100133. Advance online publication. <https://doi.org/10.1016/j.ajogmf.2020.100133> 5. Kirtsman, M., Diambomba, Y., Poutanen, S. M., Malinowski, A. K., Vlachodimitropoulou, E., Parks, W. T., et al. (2020). Probable congenital SARS-CoV-2 infection in a neonate born to a woman with active SARS-CoV-2 infection. *CMAJ* 192, E647–E650. doi:10.1503/cmaj.200821. 6. Baud, D., Greub, G., Favre, G., Gengler, C., Jaton, K., Dubruc, E., et al. (2020). Second-Trimester Miscarriage in a Pregnant Woman with SARS-CoV-2 Infection. *JAMA - J. Am. Med. Assoc.* 323, 2198–2200. doi:10.1001/jama.2020.7233. 7. Fan, C., Lei, D., Fang, C., Li, C., Wang, M., Liu, Y., Bao, Y., Sun, Y., Huang, J., Guo, Y., Yu, Y., & Wang, S. (2020). Perinatal Transmission of COVID-19 Associated SARS-CoV-2: Should We Worry?. *Clinical infectious diseases : an official publication of the Infectious Diseases Society of America*, ciaa226. Advance online publication. <https://doi.org/10.1093/cid/ciaa226> 8. Chen, H., Guo, J., Wang, C., Luo, F., Yu, X., Zhang, W., Li, J., Zhao, D., Xu, D., Gong, Q., Liao, J., Yang, H., Hou, W., & Zhang, Y. (2020). Clinical characteristics and intrauterine vertical transmission potential of COVID-19 infection in nine pregnant women: a retrospective review of medical records. *Lancet (London, England)*, *395*(10226), 809–815. <https://doi.org/10.1016/S0140-6736(20)30360-3> 9. Chen, S., Huang, B., Luo, D. J., Li, X., Yang, F., Zhao, Y., Nie, X., & Huang, B. X. (2020). *Zhonghua bing li xue za zhi = Chinese journal of pathology*, *49*(5), 418–423. <https://doi.org/10.3760/cma.j.cn112151-20200225-00138> 10. Liu, W., Wang, Q., Zhang, Q., and Chen, L. (2020). Coronavirus disease 2019 (COVID-19) during pregnancy: a case series. *Preprint* 2019, 1–28. Available at: <https://www.preprints.org/manuscript/202002.0373/v1%0Awww.preprints.org>. 11. Wang, S., Guo, L., Chen, L., Liu, W., Cao, Y., Zhang, J., & Feng, L. (2020). A case report of neonatal COVID-19 infection in China. *Clinical infectious diseases : an official publication of the Infectious Diseases Society of America*, ciaa225. Advance online publication. <https://doi.org/10.1093/cid/ciaa225> 12. Baergen, R. N., & Heller, D. S. (2020). Placental Pathology in Covid-19 Positive Mothers: Preliminary Findings. *Pediatric and developmental pathology : the official journal of the Society for Pediatric Pathology and the Paediatric Pathology Society*, *23*(3), 177–180. <https://doi.org/10.1177/1093526620925569> 13. Wang, X., Zhou, Z., Zhang, J., Zhu, F., Tang, Y., & Shen, X. (2020). A case of 2019 Novel Coronavirus in a pregnant woman with preterm delivery. *Clinical infectious diseases : an official publication of the Infectious Diseases Society of America*, ciaa200. Advance online publication. <https://doi.org/10.1093/cid/ciaa200> 14. Trippella, G., Ciarcià, M., Ferrari, M., Buzzatti, C., Maccora, I., Azzari, C., Dani, C., Galli, L., & Chiappini, E. (2020). COVID-19 in Pregnant Women and Neonates: A Systematic Review of the Literature with Quality Assessment of the Studies. *Pathogens (Basel, Switzerland)*, *9*(6), E485. <https://doi.org/10.3390/pathogens9060485> 15. Peng, Z., Wang, J., Mo, Y., Duan, W., Xiang, G., Yi, M., Bao, L., & Shi, Y. (2020). Unlikely SARS-CoV-2 vertical transmission from mother to child: A case report. *Journal of infection and public health*, *13*(5), 818–820. <https://doi.org/10.1016/j.jiph.2020.04.004> 16. Ferraiolo, A., Barra, F., Kratochwila, C., Paudice, M., Vellone, V. G., Godano, E., Varesano, S., Noberasco, G., Ferrero, S., & Arioni, C. (2020). Report of Positive Placental Swabs for SARS-CoV-2 in an Asymptomatic Pregnant Woman with COVID-19. *Medicina (Kaunas, Lithuania)*, *56*(6), E306. <https://doi.org/10.3390/medicina56060306> 17. Li, Y., Zhao, R., Zheng, S., Chen, X., Wang, J., Sheng, X....Sheng, J. (2020). Lack of Vertical Transmission of Severe Acute Respiratory Syndrome Coronavirus 2, China. *Emerging Infectious Diseases*, *26*(6), 1335-1336. https://dx.doi.org/10.3201/eid2606.200287. |
| --- |
